# RNA-seq analysis reveals a positive role for NGF in the myogenic differentiation of bovine skeletal muscle satellite cells

**DOI:** 10.3389/fgene.2025.1713817

**Published:** 2026-01-21

**Authors:** Xin Li, Zhongli Zhao, Yang Cao, Yumin Zhao, Lihong Qin

**Affiliations:** Institute of Animal and Veterinary Medicine, Ji Lin Academy of Agricultural Sciences, Gong Zhuling, China

**Keywords:** NGF, bovine skeletal muscle satellite cells, RNA-seq, myogenic differentiation, PI3K/Akt signalling pathway

## Abstract

In this study, we successfully isolated and cultured bovine skeletal muscle satellite cells (bSMSCs) and induced muscle cell formation *in vitro*. Skeletal muscle satellite cells (SMSCs) were isolated from the deep tissues of foetal bovine hind limbs and differentiated with 2% horse serum *in vitro*. The transcriptome sequencing results revealed a total of 1030 differentially expressed genes (DEGs) in the middle stage of differentiation (day 3) compared with the predifferentiation stage (day 0). A total of 374 DEGs were identified in the postdifferentiation stage (day 7) compared with the middle differentiation stage (day 3). We further investigated the regulatory effects of the DEG nerve growth factor (NGF) on the proliferation and myogenic differentiation of bSMSCs. The overexpression of NGF increased the mRNA and protein expression levels of myosin heavy chain (MyHC) and myogenin (MyoG), which are myoblast development markers, whereas NGF knockdown had the opposite effect; however, NGF did not affect the expression of the proliferation marker paired box gene 7 (Pax7) in bSMSCs. In addition, functional enrichment analysis of the DEGs revealed that the PI3K/Akt signalling pathway was significantly enriched in the DEGs and that NGF regulates myogenesis through the activation of the PI3K/Akt signalling pathway. Our results revealed that NGF was shown to be a putative regulator that controls myogenesis by activating the PI3K/Akt signalling pathway. The study provided a reference for further studies on the molecular mechanism of myogenic differentiation, regulatory network establishment, and beef quality improvement.

## 1 Introduction

The growth and development of bovine skeletal muscle can affect beef quality by altering the colour, water storage capacity, and intramuscular fat content of beef (Meng, 2022). Therefore, elucidating the regulatory mechanisms of bovine skeletal muscle growth and development (Fu et al., 2021) is highly important for improving beef quality. The maintenance of skeletal muscle tissue depends on satellite cells located close to muscle fibres. Muscle satellite cells are a heterogeneous group of cells, and a small number of them are muscle stem cells, called satellite stem cells (Dumont et al., 2015), which are responsible for muscle repair and regeneration after injury in adult animals (Peng and Huard, 2004; Chen and Goldhamer, 2003). Bovine skeletal muscle satellite cells (bSMSCs) are multifunctional special stem cells with self-renewal and myogenic abilities that can repair injured skeletal muscle, and their number gradually decreases with increasing age (Dayton and WhiteM, 2014). Skeletal muscle satellite cells (SMSCs) are distributed in the middle of the muscle membrane and basement membrane and are the only source of myogenic stem cells, which are generally in a resting state (Mauroa, 1961; Sousa-victor et al., 2022). When these cells are activated by inflammatory mediators produced in muscle tissue after injury, they are transformed into biological muscle precursor cells to form new myotubes to repair damaged muscle. The cells differentiate, proliferate, and fuse to form multinucleated cells. Finally, new muscle fibres are generated (Bokar et al., 1997; Wang W. et al., 2024).

According to previous studies, SMSCs are crucial for the regeneration and repair of skeletal muscle fibres following injury (Song and Sadayappan, 2020), but the epigenetic mechanisms regulating the fate of SMSCs and their differentiation during muscle regeneration are not fully understood (Huang et al., 2019). Skeletal muscle paralysis is caused by denervation. Previous studies have shown that NGF promotes the growth of neurons and affects the recovery of nerve function after spinal cord injury (Zou et al., 2018; Ma et al., 2018). In the clinic, nerve growth factor (NGF) is injected into damaged nerve sites to improve the motor function of patients (Camerino et al., 2018). NGF repairs nerve fibres, which promotes skeletal muscle regeneration. Studies have shown that the structural integrity and function of atrophic muscles improve after NGF is injected into the sciatic nerves of rats (Jiao et al., 2017). The aim of this study is to elucidate the mechanism through which exogenous NGF affects the proliferation and myogenic differentiation of bSMSCs *in vitro*.

We isolated bSMSCs, created a model of myogenic differentiation, and compared the transcriptomes of predifferentiation (day 0), middifferentiation (day 3), and postdifferentiation (day 7) bSMSCs via RNA sequencing (RNA-seq) to identify the genes and pathways involved in the regulation of myogenic differentiation and the effects of NGF on myogenic differentiation. We also investigated the role of NGF in myogenesis. The obtained transcriptomic data expand our knowledge of the myogenesis process, offer useful resources for the study of bovine bSMSC myogenic differentiation, and establish a strong basis for future research into the molecular mechanisms of bSMSC myogenic differentiation and improvement of beef quality.

## 2 Materials and methods

### 2.1 *In vitro* culture and differentiation of bSMSCs

The bSMSCs used in the experiments were derived from newborn Chinese Red Cattle calves and stored in the Laboratory of Cell Biology, Institute of Animal and Veterinary Medicine, Jilin Academy of Agricultural Sciences.

*In vitro*, bSMSCs were isolated, purified, cultured, and verified using previously described techniques (Li et al., 2024). Following resuspension, bSMSCs were passaged to the third generation via inoculation in T25 cell flasks. When the cells reached 60%–70% confluence, the basic medium (Dulbecco’s modified Eagle’s medium/Nutrient Mixture F-12 (DMEM/F12) +10% foetal bovine serum (FBS) +1% penicillin‒streptomycin) was replaced with myogenic differentiation medium (DMEM/F12 + 2% horse serum (HS). For the next 7 days, the induction process was continuous, and the differentiation medium was changed every 2 days.

### 2.2 RNA isolation, library preparation and sequencing

Total RNA was extracted from the bSMSCs before (day 0), during (day 3), and after (day 7) myogenic differentiation with TRIzol (Invitrogen, United States) according to the manufacturer’s instructions. To analyse the RNA expression patterns, the samples were sent to Ji Diao Co., Ltd., China. An RNA-seq library was constructed and sequenced on the basis of the Illumina HiSeq X Ten™ protocol, and 150-bp-long reads were generated. The quality of the RNA-seq reads in all the samples was examined using FastQC (0.10.1; Babraham Institute, Cambridge, United Kingdom). These reads were mapped to the *Bos taurus* genomes that passed quality control (2.1.0).

### 2.3 Verification of differentially expressed genes (DEGs)

We identified DEGs (IGF1, FGF10, COL6A1, EREG, and NGF) involved in myogenic differentiation and verified their expression via qRT‒PCR. GAPDH was used as the internal control. Total RNA was isolated from predifferentiation (day 0), middifferentiation (day 3), and postdifferentiation (day 7) cells and reverse-transcribed into cDNA in three replicates per group. To perform qRT‒PCR, the cDNA was diluted five times with ddH_2_O. The primers were synthesized by Shanghai Shenggong Bioengineering Company, and the sequences are shown in Table 1. The PCR mixture was as follows: SYBRTaq, 10 μL; cDNA, 2 μL; upstream and downstream primers, 0.4 μL; ddH_2_O supplementation to a total volume of 20 μL. The reaction procedure was 95 °C for 2 min, 95 °C for 3 s, and 60 °C for 30 s, for a total of 40 cycles; 95 °C for 15 s, 65 °C for 1 min, and 95 °C for 15 s.

**TABLE 1:** Primer information of DEGs for qRT‒PCR.

Genes	Primer sequences (5′–3′)	Product length (bp)	Annealing temperature (°C)
IGF1	F: GCT​TTT​GTG​ATT​TCT​TGA​AGC​AG	355	60
	R: TTC​TTC​AAA​TGT​ACT​TCC​TTC​TGA​G		
FGF10	F: GTG​TCT​TCC​GTC​CCT​GTC​AC	239	60
	R: ATC​TCC​AGG​ATA​CTG​TAC​GGG		
COL6A1	F: CAT​CAC​CAA​ACG​CTT​TGC​CA	198	60
	R: TCG​GTG​GCG​TCA​TTG​AAG​AA		
EREG	F: TGT​ATC​CCA​GGA​GAG​TCG​GG	196	60
	R: AGA​AAT​GCT​CAC​ACC​GGA​CA		
NGF	F: ACA​TCA​AGG​GCA​AGG​AGG​TG	243	60
	R: CAG​TCT​TCC​TGC​TGA​GCA​CA		
GAPDH	F: GTC​GGA​GTG​AAC​GGA​TTC​GG	238	60
	R: CCA​GCA​TCA​CCC​CAC​TTG​AT		

### 2.4 siRNA knockdown and overexpression plasmid DNA construction and transfection

The mRNA sequences of three RNAi target sites, 482, 608, and 193, were chosen using the reference bovine NGF gene sequence (NM_001099362.1) to construct plasmids. The plasmid sequences are displayed in Table 2. A noncoding sequence was used to construct a negative control plasmid, and Shanghai Gima Pharmaceutical Technology Co., Ltd., created three target plasmids, which were termed NGF1-BOS-482, NGF-BOS-608, and NGF-BOS-193.

**TABLE 2:** siRNA sequences of the NGF1 gene.

siRNA	Primer sequences (5′–3′)
Negative control	CAC​TCT​GAT​CAC​AGC​TCT​TTT​GA
	AAG​TTT​AAT​CCA​GTG​GGC​TTG​AG
NGF-BOS-482	GAG​AGG​UGA​ACA​UCA​ACA​ATT
	UUG​UUG​AUG​UUC​ACC​UCU​CTT
NGF-BOS-608	CGA​CCC​ACA​CCU​UCG​UCA​ATT
	UUG​ACG​AAG​GUG​UGG​GUC​GTT
NGF-BOS-193	GGG​CAG​ACC​CAC​AAC​AUC​ATT
	UGA​UGU​UGU​GGG​UCU​GCC​CTT

The CDS region-amplified fragment of the NGF mRNA sequence (NM_001099362.1) was cloned and inserted into the overexpression vector pcDNA3.1 (+).

A total of 5 × 10^5^ cells were inoculated in a six-well plate, and the overexpression plasmid and knockdown plasmid were transfected into liposomes. One hundred microlitres of serum-free Opti-MEM was diluted to 100 nM, mixed gently, and incubated at room temperature for 5 min (a); 3 μL of Lipofectamine 3000 was diluted with 100 μL of serum-free Opti-MEM, gently mixed and incubated at room temperature for 5 min (b). a and b were gently mixed and incubated at room temperature for 10 min, and then 200 μL of the liposome transfection mixture was added to each well of a 6-well plate and cultured at 37 °C in a 5% CO_2_ incubator. After 48 h of culture, the complete medium was replaced, and the culture was continued. After the transfection protocol was complete, the transfected cells were incubated in myogenic differentiation medium for 2 days.

### 2.5 qRT‒PCR

The mRNA expression levels of the NGF gene, the marker genes MyHC and MyoG, and the PI3K and Akt genes before and after differentiation were measured via qRT‒PCR. Total RNA was extracted from predifferentiated (day 0) and postdifferentiation (day 7) cells, with 3 replicates per group, and reverse-transcribed into cDNA. The reverse transcription product was diluted 5 times with ddH_2_O and used for qRT‒PCR. The primers were synthesized by Shanghai Shenggong Bioengineering Company, and the primer sequences are shown in Table 3. The PCR mixture and the reaction procedure were the same as those described in Section 2.4.

**TABLE 3:** Primer information for qRT‒PCR.

Genes	Primer sequences (5′–3′)	Product length (bp)	Annealing temperature (°C)
MyHC	F: AAG​CTG​ATG​CCT​TGG​CTG​AT	219	60
	R: TCT​CTG​TGG​CGT​GTT​TCT​CC		
MyoG	F: CAG​TAC​ATA​GAG​CGC​CTG​CA	235	60
	R: TCC​ACT​GTG​ATG​CTG​TCC​AC		
PI3K	F: CTA​TCC​TGT​GCC​GGC​TAC​TG	265	60
	R: CCA​TGC​CGG​CGT​AAA​ATC​AG		
Akt	F: CAT​GCA​GCA​CCG​ATT​CTT​CG	201	60
	R: CGA​GTA​GGA​GAA​CTG​GGG​GA		

### 2.6 Western blot analysis

The protein samples were separated via 12% SDS‒PAGE, lysed with RIPA buffer, and transferred to PVDF membranes. Primary antibodies against NGF(Bioss, bs-10806R, Beijing, China, 1:2000 dilution), PAX7 (DSHB, Pax7, United States, 1:1500 dilution), MyoD1(Bioss, bs-62914R, Beijing, China, 1:1500 dilution), MyHC(DSHB,10F5, United States, 1:1500 dilution), MyoG (Bioss, bs-3550R, Beijing, China, 1:1500 dilution), Akt (CST, 9272S, United States, 1:2000 dilution), p-Akt (CST, 9271S, United States,1:2000 dilution), PI3K(CST, 4249S, United States, 1: 2000 dilution), p-PI3K(CST, 4228S, United States, 1: 2000 dilution)and GAPDH(Proteintech, 60004-1-Ig, United States, 1: 5000 dilution) were diluted with TBST, and the total protein concentrations of the samples were determined using a BCA assay.

Following an overnight incubation period at 4 °C with the primary antibodies, the samples were incubated for 1 h at room temperature with either anti-rabbit or anti-mouse IgG HRP-linked antibodies. The proteins were then visualized using enhanced chemiluminescence (ECL) hypersensitive solution.

### 2.7 Statistical analysis

All the data are expressed as the standard deviation ±mean. All statistical studies were significant at p < 0.05. For each statistically significant result, one-way ANOVA and *post hoc* tests were performed using GraphPad Prism 7 (GraphPad Prism software, Inc., La Jolla, CA, United States). The results from three independent experiments were averaged.

## 3 Results

### 3.1 Isolation and identification of bSMSCs

**FIGURE 1:**
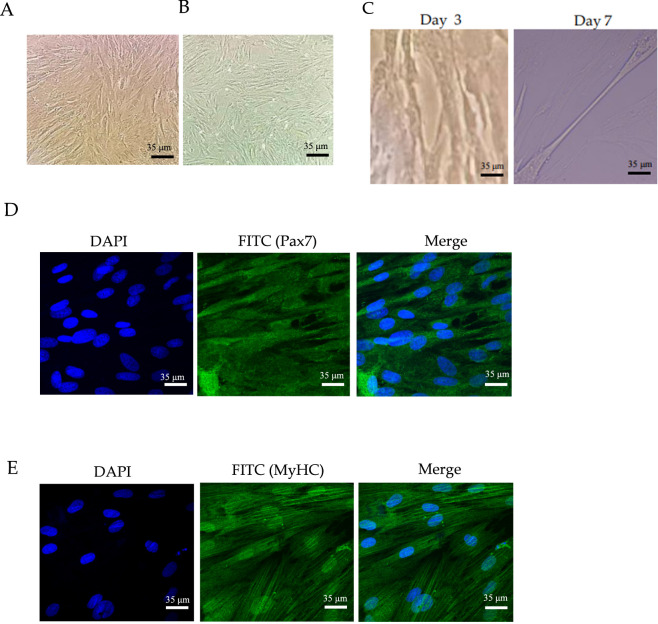
Isolation and identification of bSMSCs. **(A)** Primary bSMSCs cultured for 5–7 days. **(B)** The third generation of bSMSCs. **(C)** bSMSCs at the 3rd and 7th days of myogenic differentiation. **(D)** Immunofluorescence staining of specific marker Pax7 for SMSCs. **(E)** Immunofluorescence staining of MyHC in bSMSCs on the 7th day.

The isolated bSMSCs were small in size and grew in clusters. With increasing passage number, the cells gradually became confluent. The bottom of the dish was fully covered with cells after 5–7 days of cultivation (Figure 1A). When the cells were passaged to the second day of the third passage, the morphology of the bSMSCs was relatively stable; most of the cells presented a long and fusiform morphology, while some were polygonal in shape (Figure 1B). During the process of myogenic differentiation, bSMSCs gradually become thicker and more mature as the differentiation time prolongs. On the 7th day, new muscle tubes can be observed under the microscope (Figure 1C). The results of immunofluorescence staining showed that the specific markers Pax7 of SMSCs were positively expressed, indicating that the cells we isolated were bSMSCs (Figure 1D). After 7 days of bSMSC myogenic induction, the newly formed myotubes were observed under a microscope. Immunofluorescence revealed positive expression of the myogenic differentiation marker MyHC, indicating that bSMSCs could fuse into muscle ducts (Figure 1E).

### 3.2 Transcriptome library preparation and sequencing

The third-generation bSMSC transcriptome was sequenced using the Illumina X plus platform. After low-quality data from the predifferentiation stage (day 0), middifferentiation stage (day 3), and late differentiation stage (day 7) were eliminated, high-quality clean data that contained 11.5G, 11.8G, and 11.7G nucleotides, respectively, and data that contained poly-N or adaptors were obtained. For all of these samples, the Q20% scores (error identification ratio of nucleotides with a Phred quality score <0.01) of the clean data were greater than 97%, suggesting high-quality sequencing data. The GC content (%) of the gene sequences obtained from cells in the predifferentiation, middifferentiation, and postdifferentiation stages was 46.73%, 48.95%, and 46.37%, respectively (Table 4; Supplementary Figure S1). The sequencing saturation of the transcriptome was evaluated using saturation curves. When the saturation curve reached a plateau, we assessed it as sequencing saturation.

**TABLE 4:** Quality of sequencing.

Sample	Raw data (G)	Clean data (G)	Q20 (%)	Q30 (%)	GC content (%)
Day-0	11.6	11.5	97.82	93.51	46.73
Day-3	11.9	11.8	97.41	92.75	48.95
Day-7	11.7	11.7	97.42	92.61	46.37

### 3.3 DEG screening and validation

**FIGURE 2:**
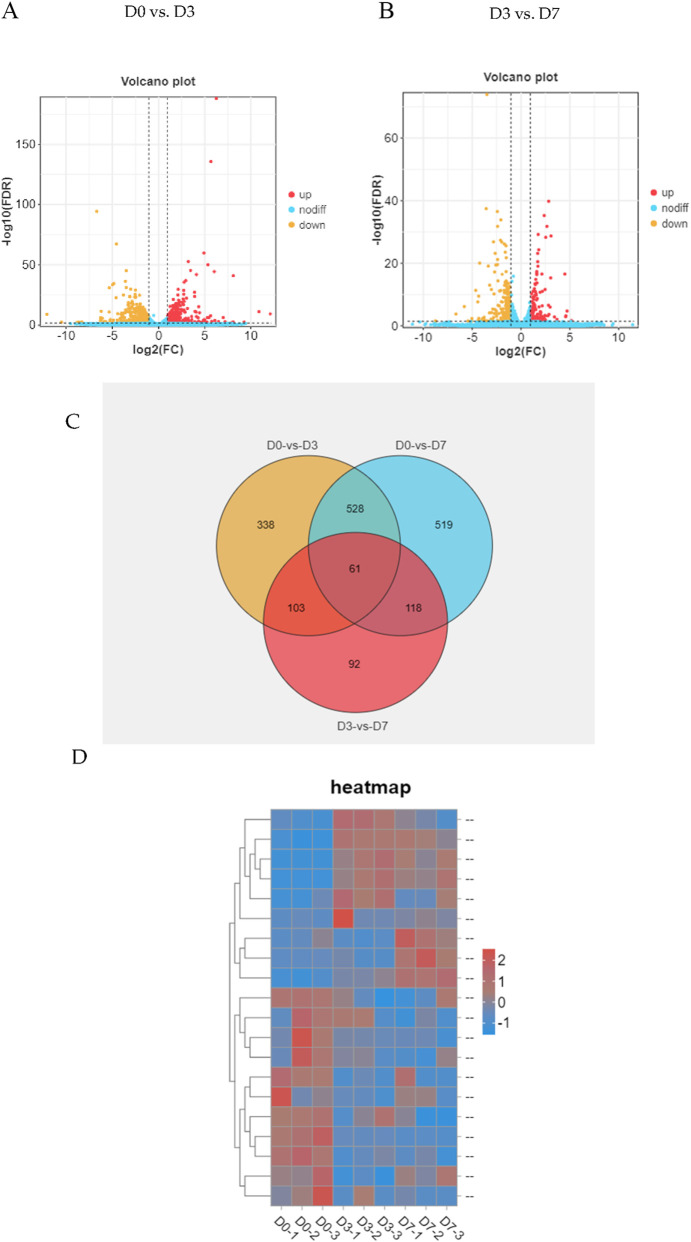
Comparison of DEGs identified during several myogenic differentiation stages. **(A)** Volcano plot analysis of differentially expressed genes (DEGs) in the early stage of myogenic differentiation; **(B)** Volcano plot analysis of DEGs in the later stage of myogenic differentiation; **(C)** Venn diagram analysis of DEGs in various stages of myogenic differentiation; **(D)** Heatmaps of DEGs in various stages of myogenic differentiation.

**FIGURE 3:**
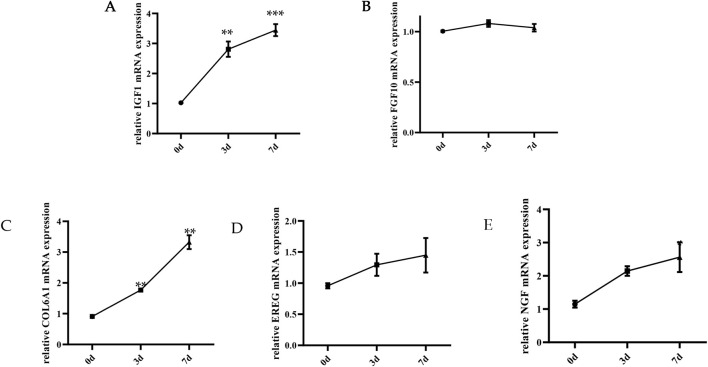
qRT‒PCR validation of the DEGs from RNA sequencing. **(A)** Insulin-like growth factor 1 (IGF1); **(B)** Fibroblast growth factor 10 (FGF10); **(C)** Collagen type VI alpha 1 chain (COL6A1); **(D)** Epiregulin (EREG); **(E)** nerve growth factor (NGF).*p < 0.05; **p < 0.01; ****p < 0.001.

After significance and fold change (p < 0.05, log2-fold change > |1|) were set, DEGseq was used for differential expression analysis. Compared with those on day 0, a total of 1030 DEGs (including several presumed new genes) were identified on day 3; 412 genes were upregulated, and 618 genes were downregulated (Figures 2A,B). A total of 374 DEGs, including several presumed novel genes, 161 upregulated and 213 downregulated, were identified on day 7 compared with day 3. A thorough analysis of the data revealed that 61 genes were expressed differently in skeletal muscle satellite cells during each of the three stages of myogenic differentiation (Figures 2C,D). To verify these DEGs, we selected 5 genes and evaluated their expression patterns during myogenic differentiation via qRT‒PCR and compared them with the RNA-seq results; these genes included insulin-like growth factor 1 (IGF1), fibroblast growth factor 10 (FGF10), collagen type VI alpha 1 chain (COL6A1), epiregulin (EREG), and nerve growth factor (NGF). These 5 genes are located in the PI3K/AKT signalling pathway, and their TPM (transcripts per million, TPM) values significantly changed according to the results of the RNA-seq analysis (Supplementary Table S1). The findings demonstrated that the qRT‒PCR data matched the RNA-seq data, confirming the high reliability of the DEGs identified in this investigation (Figures 3A–D). The results of the qRT‒PCR verification indicated that the expression of the FGF10 and EREG genes did not significantly differ during myogenic differentiation, whereas the expression of the IGF1, COL6A1, and NGF genes significantly differed during this process. Given the size of the CDS region sequences of these three genes and the convenience of constructing overexpression plasmids later for studying the functions of the genes, the NGF gene was ultimately selected for subsequent research.

### 3.4 Gene Ontology (GO) and Kyoto Encyclopedia of Genes and Genomes (KEGG) analyses of the DEGs

**FIGURE 4:**
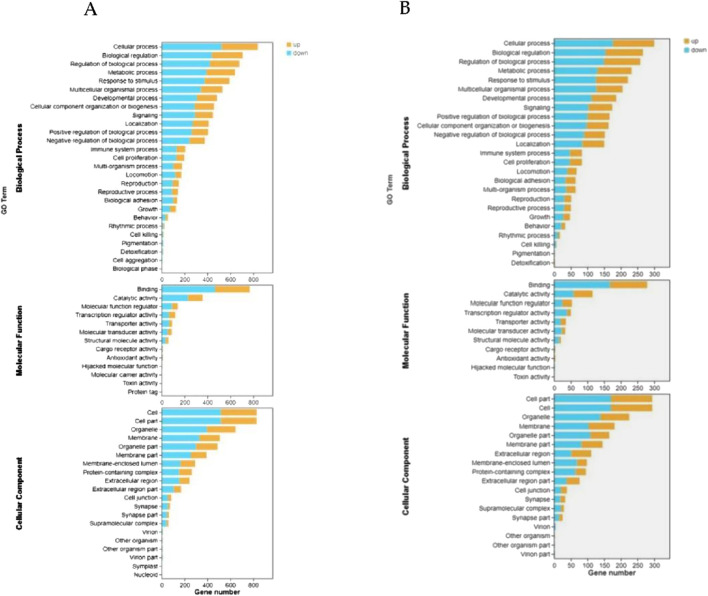
Gene Ontology (GO) annotations of the transcriptome unigenes. The figure is divided into three sections: cellular components, molecular functions, and biological processes. The significance level of enrichment was set at a corrected *p* value (*p* < 0.05). **(A)** GO annotations of unigenes in the early stage (D0-D3) of myogenesis; **(B)** GO annotations of unigenes in the later stage (D3-D7) of myogenesis.

**FIGURE 5:**
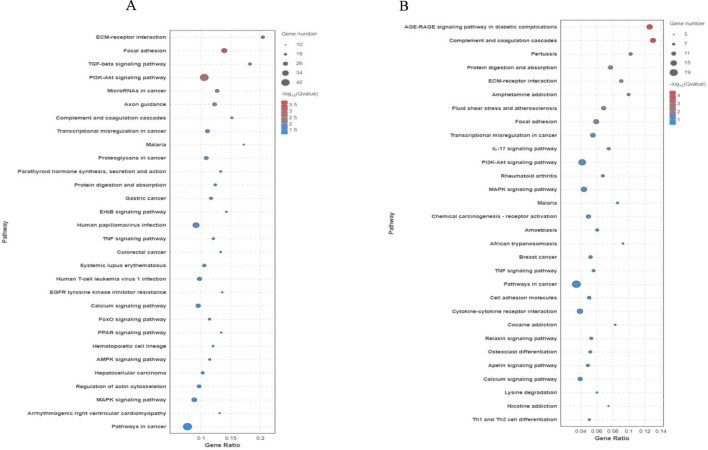
Kyoto Encyclopedia of Genes and Genomes (KEGG) functional annotations of unigenes in the transcriptome. The X-axis label represents the gene ratio (gene ratio = number of DEGs enriched in the pathway/number of all genes in the annotated gene set), and the Y-axis label represents the pathway (*p* adjusted <0.05). **(A)** KEGG of unigenes in the early stage (D0–D3) of myogenesis; **(B)** KEGG of unigenes in the later stage (D3–D7) of myogenesis.

**FIGURE 6:**
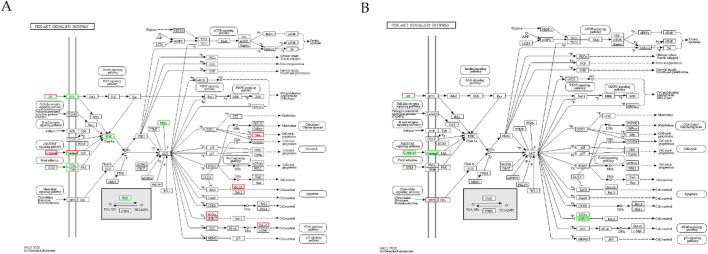
PI3K/Akt signalling pathway of unigenes in the transcriptome. **(A)** PI3K/Akt signalling pathway of unigenes in the early stage (D0–D3) of myogenesis; **(B)** PI3K/Akt signalling pathway of unigenes in the later stage (D3–D7) of myogenesis.

We conducted GO and KEGG pathway enrichment analyses of DEGs on days 0, 3, and 7 after establishing the significance level (p < 0.05) to understand the biological functions of the DEGs in SMSCs. A GO enrichment histogram was constructed to visualize the enriched biological process, molecular function, and cellular component terms among the DEGs. The enriched biological processes were cellular processes and biological process regulation. Many DEGs were associated with the molecular function terms binding, catalytic activity, and molecular function regulator. Finally, the enriched cellular component terms were cell part, cell, and organelle (Figures 4A,B). KEGG pathway analysis revealed that the phosphoinositide 3-kinase (PI3K)-Akt signalling pathway, pathways related to cancer, the MAPK signalling pathway, and pathways related to human papillomavirus infection were enriched mainly in the DEGs (Figures 5A,B). Studies have shown that the PI3K/AKT signalling pathway plays a crucial role in muscle generation and the formation of new blood vessels (Zhou et al., 2007; Bian et al., 2010). The PI3K/Akt signalling pathway was the most enriched pathway among the DEGs of bSMSCs at various stages of SMSC differentiation (Figures 6A,B).

### 3.5 Analysis of NGF gene expression during myogenic differentiation

**FIGURE 7:**
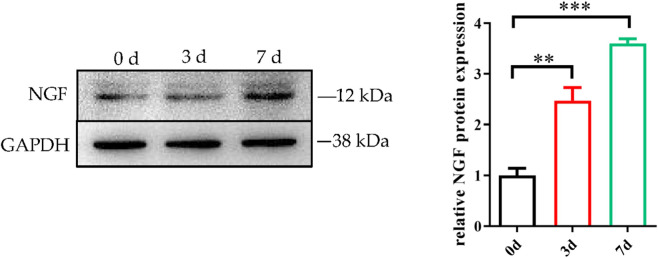
Role of NGF in the regulation of myogenic differentiation. NGF mRNA expression on days 0, 3, and 7, as shown in Figure 3E. NGF protein expression on days 0, 3, and 7. **p < 0.01; ***p < 0.001.

Previous studies have shown that NGF is a key regulator of satellite cell myogenic differentiation and has a protective effect on myogenic differentiation. However, the exact functions of NGF, such as its effects on the proliferation, survival, differentiation, and signalling of myogenic cells, remain unclear (Alessandra et al., 2017). On days 0, 3, and 7 of myogenic differentiation, the levels of NGF mRNA and protein expression were assessed using Western blotting and qRT‒PCR. The findings demonstrated that NGF expression was upregulated on day 3 and highlighted significant expression differences on day 7 compared with day 0 (Figures 3E, 7); thus, NGF was selected for functional analysis.

### 3.6 Determination of the effect of plasmid and siRNA transfection on NGF gene overexpression and knockdown

**FIGURE 8:**
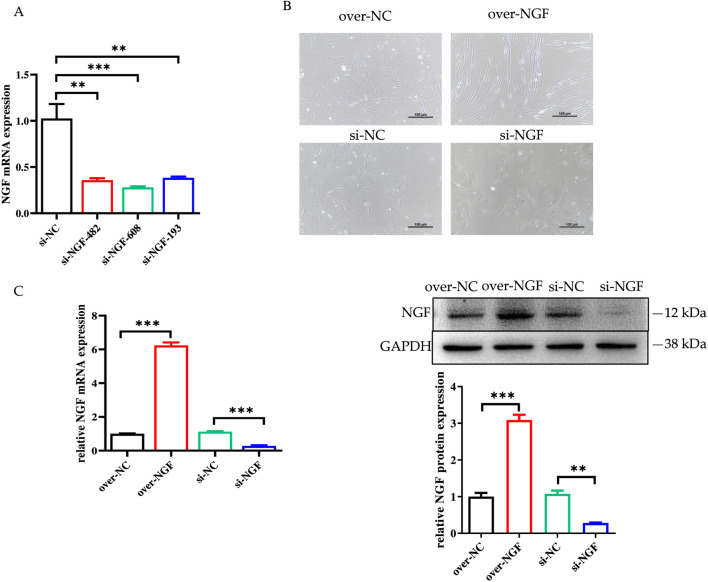
Determination of the effects of plasmid and siRNA transfection on NGF gene overexpression and knockdown. **(A)** NGF mRNA expression levels after transfection with the three NGF siRNAs. **p < 0.01; ***p < 0.001. **(B)** The transfection efficiency of NGF overexpression and knockdown; **(C)** The expression level of NGF after NGF overexpression and knockdown.

Using siRNA knockdown and overexpression techniques, we examined the effects of NGF gene expression on the proliferation and myogenic differentiation of bSMSCs. We constructed three siRNAs to transfect bSMSCs using the negative control. The transfection effects are shown in Figure 8A. Compared with the other groups, the NGF-BOS-608 group demonstrated the best silencing effect, the best transfection effect, and the lowest NGF mRNA expression. As a result, NGF-BOS-608 was chosen for the subsequent examination.

After cells were infected with the NGF overexpression plasmid and knockdown siRNA for 48 h, microscopic observation revealed that the number of cells in the overexpression group increased significantly, while the number of cells in the knockdown group decreased significantly (Figure 8B), indicating that NGF overexpression and knockdown via infection were successful. Moreover, the mRNA and protein expression levels of NGF in the NGF-overexpressing group were significantly greater than those in the control group, whereas those in the NGF-knockdown group were significantly lower than those in the control group (Figure 8C). The results showed that the NGF gene positively regulated the myogenic differentiation of bSMSCs.

### 3.7 Role of NGF in the regulation of the proliferation and myogenic differentiation of bSMSCs

**FIGURE 9:**
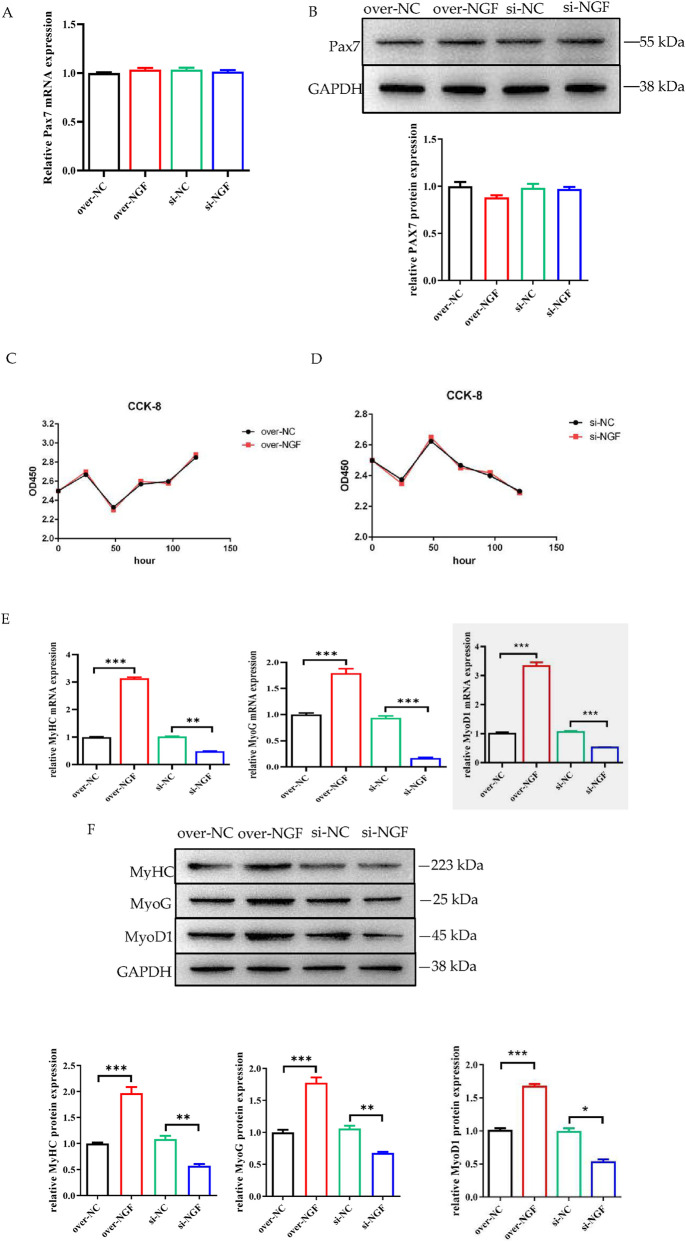
Role of NGF in the regulation of myogenic differentiation. **(A)** Determination of the mRNA levels of Pax7, a marker gene of proliferation. **(B)** Determination of the protein levels of Pax7. **(C)** Detection of the effect of the over-NGF on the proliferation of bSMSCs using the CCK8 method. **(D)**Detection of the effect of the si-NGF on the proliferation of bSMSCs using the CCK8 method. **(E)** Determination of the mRNA expression of MyHC, MyoG and MyoD1-myogenic differentiation marker genes. **(F)** Determination of the protein expression of MyHC, MyoG and MyoD1. **p < 0.01; ***p < 0.001.

The effects of NGF on the expression of the proliferation marker gene Pax7 in bSMSCs were detected. The findings revealed that the expression of paired box gene 7 (Pax7), a proliferative marker gene of bSMSCs, was not affected by NGF overexpression or interference after induction, either in terms of mRNA or protein expression (Figures 9A,B). After over-NGF and si-NGF transfection of bSMSCs, the effect of the NGF gene on cell proliferation was detected using the CCK-8 method. The results showed that the change in NGF expression level had no effect on the proliferation of bSMSCs (Figures 9C,D). The results revealed that the NGF gene did not affect the proliferation of bSMSCs.

Following the extraction of total RNA and protein from differentiated cells, the expression levels of the myogenic differentiation marker genes MyHC, MyoG and MyoD1 were assessed via qRT‒PCR and Western blotting. Compared with those in the control group, the mRNA and protein expression levels of the myogenic differentiation markers MyHC, MyoG and MyoD1 were significantly greater in the group with induced NGF overexpression. These findings suggest that NGF overexpression may have a major positive effect on these indicators. The overexpression of the NGF gene greatly increased the mRNA and protein expression of the myogenic differentiation markers MyHC, MyoG and MyoD1. In contrast, knockdown of NGF expression significantly reduced the mRNA and protein expression of MyHC and MyoG (Figures 9E,F).

### 3.8 NGF positively regulates myogenic differentiation via the PI3K/Akt signalling pathway

**FIGURE 10:**
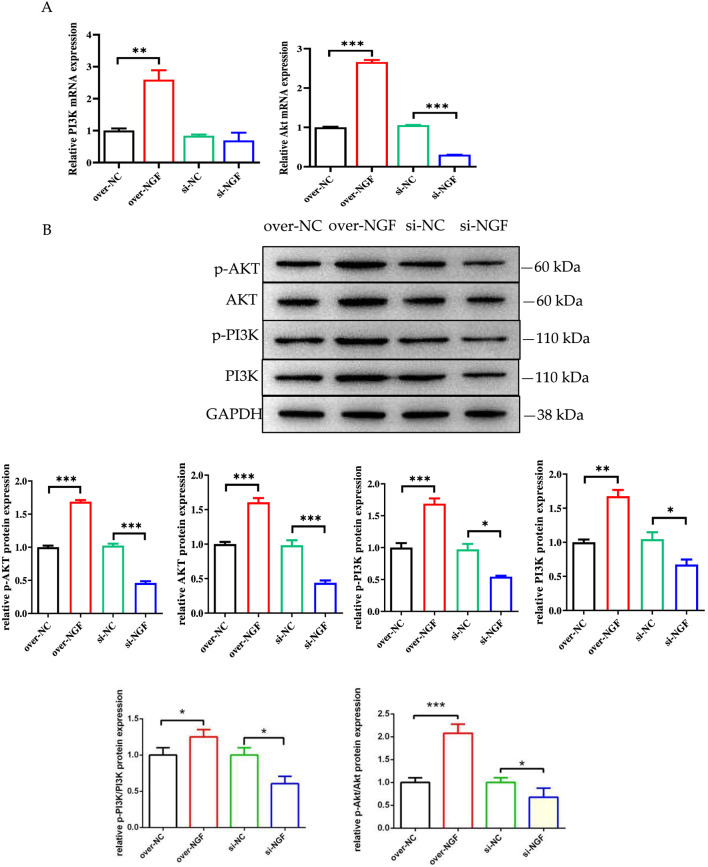
NGF affects genes, protein expression, and phosphorylation related to the PI3k/AKT signalling pathway. **(A)** Expression levels of the PI3K and AKT genes. **(B)** Expression levels of the PI3K, p-PI3K, AKT, p-AKT, p-PI3K/PI3K and p-AKT/AKT proteins. The level of total GAPDH was used as a loading control. *p < 0.05; **p < 0.01; ***p < 0.001.

The PI3K/AKT signalling pathway is important for the myogenic differentiation of bSMSCs. We investigated the effects of the knockdown or overexpression of the NGF gene on PI3K/Akt phosphorylation during the myoblastic differentiation of bSMSCs. In the process of total protein extraction, protease and phosphatase inhibitors were added to the protein lysate in RIPA buffer to ensure that the phosphorylation signal could be detected and preserved. The Western blot results are shown in Figure 8. We discovered that Akt expression strongly increased (P < 0.001) in the NGF-overexpressing group but strongly decreased (P < 0.001) in the NGF-knockdown group. NGF overexpression strongly increased Akt phosphorylation at Ser473 (P < 0.001), whereas NGF knockdown significantly inhibited Akt phosphorylation at Ser473 (P < 0.001), as indicated by the anti-phospho-Akt Ser473 antibody (Figures 10A,B).

## 4 Discussion

When satellite cells are activated, they proliferate via either symmetrical division or asymmetrical division. These cells, through proliferation, mutual fusion or differentiation, subsequently repair damaged muscle fibres and restore muscle structural integrity and function (Dumont et al., 2015; Sousa-Victor et al., 2022; Liu et al., 2024). The proliferation and differentiation of satellite cells are not only affected by myogenic regulatory factors but also regulated by many transcription factors, and the activation or inhibition of various signalling pathways also has different effects on satellite cells. These factors are indispensable for the growth and development of skeletal muscle and are crucial for muscle cell proliferation and differentiation and the formation of muscle fibres (Schiaffino et al., 2013; Relaix et al., 2021; Wang S. C. et al., 2022; Wang Z. Y. et al., 2024).

Myogenic differentiation is regulated by multiple cellular signalling pathways, including protein kinases activated by promyogenic stimuli and reversible phosphorylation. As an intracellular phosphatidylinositol kinase, PI3K is an important signal transduction molecule in cells. It is involved in the regulation of many physiological processes, such as cell proliferation, apoptosis, and differentiation. By producing phospholipids that activate serine/threonine protein kinases (Akt) and other downstream effectors, PI3K transduces signals from various growth factors and cytokines to intracellular messengers. According to earlier research, the PI3K/Akt pathway is an essential signal transduction route that controls cell adhesion, proliferation, and differentiation (Smith et al., 1988; Cornelius et al., 1994). It also plays a critical role in nerve regeneration by affecting cell growth and survival. Additionally, under stage-specific circumstances, the PI3K/AKT pathway functions as an effector of many stimuli that can induce myogenic differentiation. For example, by activating the IGF-1/PI3k/Akt/FoxO3a phosphorylation pathway, glucagon-like peptide-2 (GLP-2) significantly reverses the declines in muscle weight, relative grip strength, muscle fibre diameter, and cross-sectional area in aged mice (Ye et al., 2024). Furthermore, by influencing MyoD-regulated activity through the inhibition of PI3K/AKT activity, 4-octyl itaconate (OI) prevents muscle differentiation (Wang L. et al., 2022). Abnormal expression of genes related to the PI3K‒Akt signalling pathway or abnormal phosphorylation of proteins have been observed in the muscles of patients with muscular dystrophy and Duchenne muscular dystrophy (DMD) (Small et al., 2010; Alexander et al., 2011). In addition to DMD, other types of muscular atrophy, such as progressive proximal muscle atrophy of the limbs, myopathic facial muscle atrophy, atrophy of the interosseous muscles and the flexor digitorum muscles, and spinal muscular atrophy, have not been reported. In addition, the PI3K/Akt signalling pathway plays an important role in the maintenance of skeletal muscle (Shahini et al., 2018). Despite the extensive documentation of the promotion of myogenic differentiation by the PI3K/Akt pathway, many of its precise mechanisms are still unknown.

To investigate transcriptional information during myogenic differentiation, we performed RNA-Seq before induction (Day 0), during induction (Day 3), and after induction (Day 7). Through Gene Ontology annotations of unigenes in the transcriptome, we obtained the biological processes, molecular functions, and myogenesis differentiation of bSMSCs and were able to predict the main regulatory genes of bovine myogenesis. KEGG pathway analysis revealed that the PI3K/Akt signalling pathway was the most enriched pathway among the DEGs associated with bSMSC myogenic differentiation. We screened the 5 genes associated with myogenesis from the obtained DEGs of the PI3K/AKT signalling pathway and used qRT‒PCR to assess the reliability of the RNA-seq results. The findings revealed notable variations in the expression of the *IGF*1, *COL*6*A*1, and *NGF* genes throughout differentiation. The *NGF* gene was ultimately chosen for the subsequent investigation on the basis of the length of its CDS region sequence and the viability of creating overexpression plasmids to examine gene functions. Moreover, *FGF*10 is upregulated during early myoblastic differentiation and downregulated during late myoblastic differentiation. However, the expression of *IGF*1, *COL*6*A*1, *EREG*, and *NGF* gradually increased over time. On the basis of sequencing data from cells at various stages, we postulated that transcriptional regulators are more crucial in the early phases of myogenesis.

The members of the nerve growth factor family include nerve growth factor, brain-derived neurotrophic factor, neuronutrient-3, neuronutrient-4/5, neuronutrient-6, and neuronutrient-7, etc. NGF is one of the most important nerve growth factor proteins; it is present as a major precursor in tissues and is processed into mature NGF in submandibular glands. NGF is an endogenous protein involved in the development, maintenance, and regeneration of mammalian neurons. Given that skeletal muscle cells have significant levels of neurotrophin receptors, the neurotrophins generated by skeletal muscle may function as autocrine or paracrine factors inside the muscle tissue (Sakuma and Yamaguchi, 2011). They aid in the survival and innervation patterning of motor neurons during development, regulate muscle regeneration, and impact muscle strength (Delezie et al., 2019). According to previous reports, p75NTR may move into the nucleus upon proNGF activation and interact with adaptor proteins to control transcriptional activation, nucleocytoplasmic shuttling, and signalling cascades (Schachtrup et al., 2015; Shanab et al., 2015). Despite this evidence, nothing is known about how NGF signalling affects skeletal muscle homeostasis, and no research has thoroughly examined the potential role of NGF in controlling skeletal muscle phenotype. Crucially, to our knowledge, previous research has demonstrated only the purported biological action of mature NGF treatment (Alessandra et al., 2017). In this study, the effects of NGF on the proliferation and differentiation of myogenic precursors were evaluated.

Myogenic regulatory factors (MRFs) play important roles in myogenic differentiation. Among them, MyoD and MyoG inhibit myocyte proliferation and promote differentiation, especially in the early stage of skeletal muscle development (Pownall et al., 2002). A key marker gene for the myogenic differentiation of SMSCs is MyHC, a muscle fibre motor protein (Paula et al., 2024). In the early phases of gene myogenesis, transient RNA interference approaches can reduce gene expression by silencing specific mRNA molecules. Therefore, we analysed the function of the NGF gene via RNAi knockdown and overexpression techniques. We discovered that the NGF gene expression level increased steadily throughout myogenic differentiation, peaking on day seven. The mRNA and protein expression levels of the NGF-overexpressing group were significantly greater than those of the control group, and the mRNA and protein expression levels of the myoblast differentiation markers MyHC, MyoG and MyoD1 were significantly increased by NGF gene overexpression. Compared with those in the control group, the MyHC, MyoG and MyoD1 expression levels in the NGF knockdown group were noticeably lower. Akt expression and phosphorylation at Ser473 were markedly increased by NGF overexpression, whereas these processes were markedly inhibited by NGF knockdown.

## 5 Conclusion

In conclusion, the genes involved in the myogenesis of bSMSCs were revealed in this study. Using RNA-seq, NGF was shown to be a putative regulator that controls myogenesis by activating the PI3K/Akt signalling pathway. In our study, we obtained valuable genetic resources and sequencing information related to the process of myogenic differentiation and analysed the regulatory mechanisms of several functional genes, providing a reference for further studies on the molecular mechanism of myogenic differentiation, regulatory network establishment, and beef quality improvement.

## Data Availability

The datasets presented in this study can be found in online repositories. The names of the repository/repositories and accession number(s) can be found below: https://bigd.big.ac.cn/gsa/browse/CRA025912, Accession No.CRA025912.
