# Supplementary figures and images for: RNA-seq analysis reveals a positive role for NGF in the myogenic differentiation of bovine skeletal muscle satellite cells
Source: Front Genet. 2026 Jan 21;16:1713817. doi: 10.3389/fgene.2025.1713817 (PMC12867674; doi:10.3389/fgene.2025.1713817)

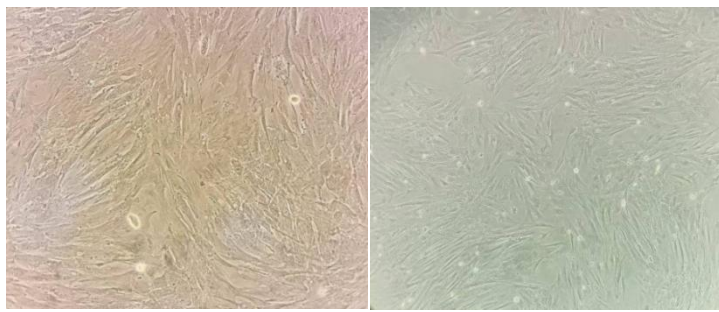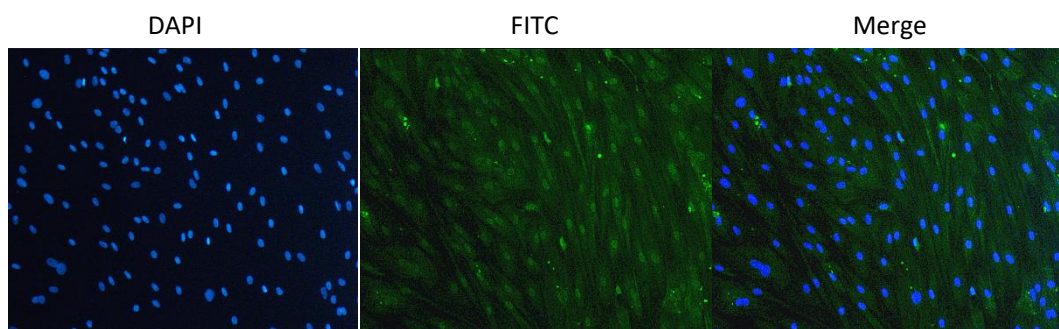

Fig.1

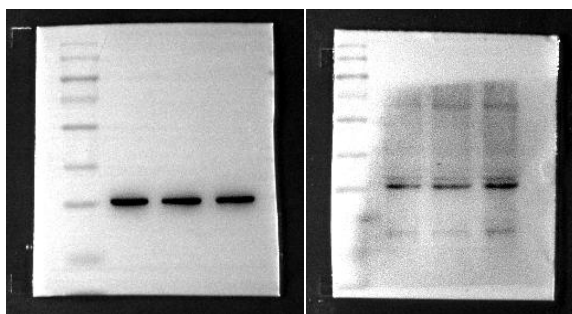

GAPDH

NGF

Fig. 7

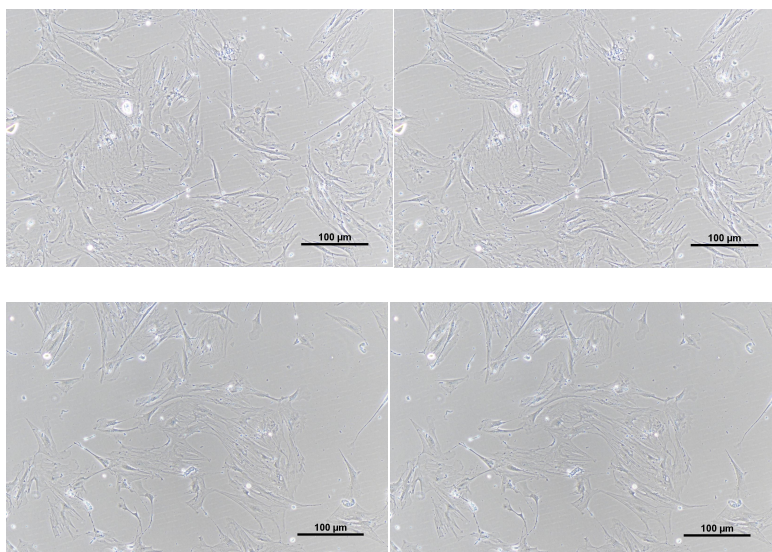

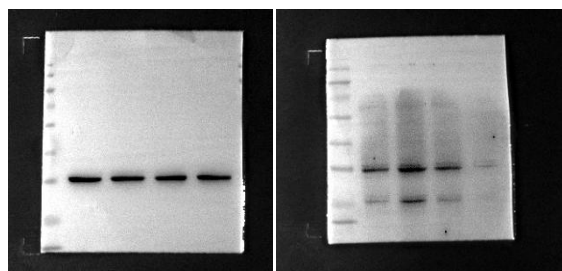

GAPDH

NGF

Fig. 8

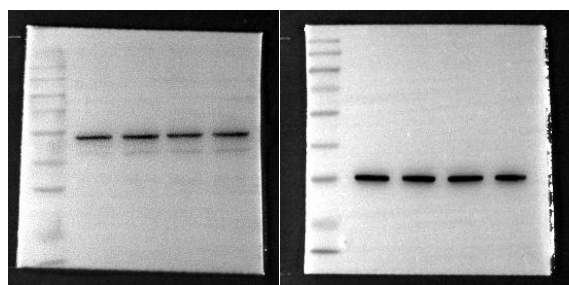

Pax7

GAPDH

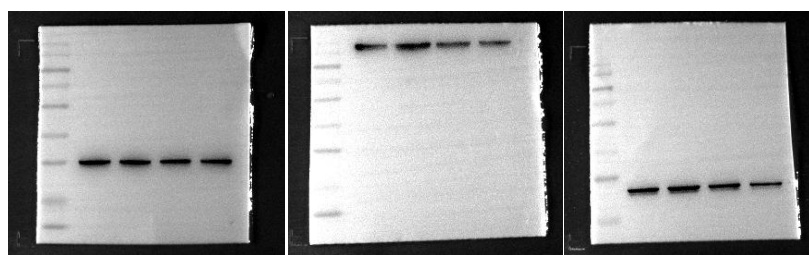

GAPDH

MyHC

MyoG

Fig.9

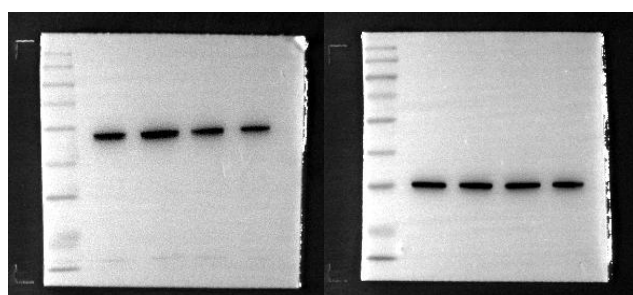

Akt

GAPDH

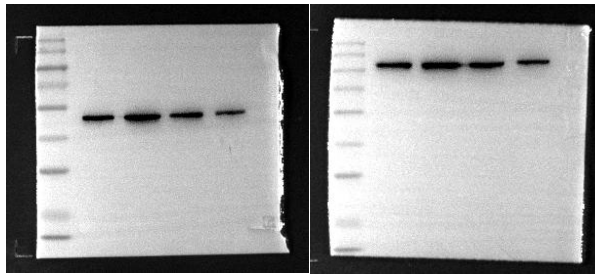

p-Akt

PI3K

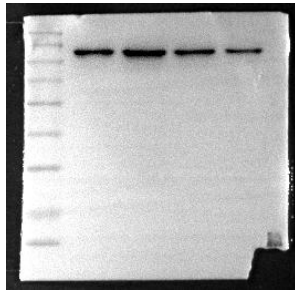

P-PI3K

Fig.10

Supplement: Supplementary file 1 [file DataSheet1.pdf]
