# Supplementary material for: RNA-seq analysis reveals a positive role for NGF in the myogenic differentiation of bovine skeletal muscle satellite cells
Source: Front Genet. 2026 Jan 21;16:1713817. doi: 10.3389/fgene.2025.1713817 (PMC12867674; doi:10.3389/fgene.2025.1713817)
Supplement: Supplementary file 2 [file DataSheet2.docx]

Supplementary Material

## Supplementary Figures

A


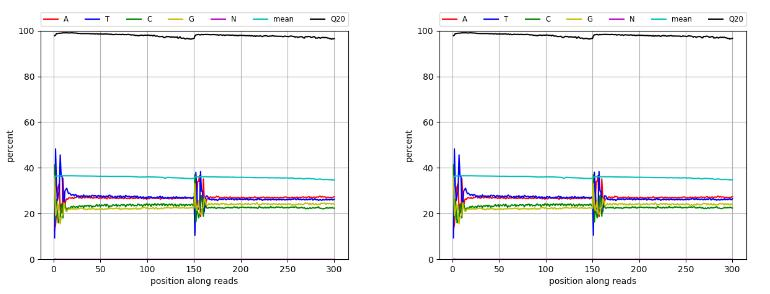


B

pre-filter after-filter


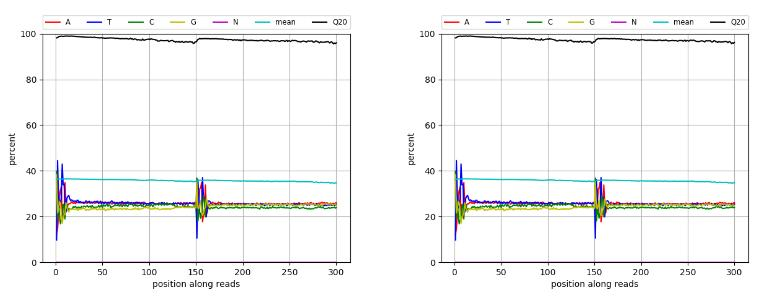


pre-filter after-filter


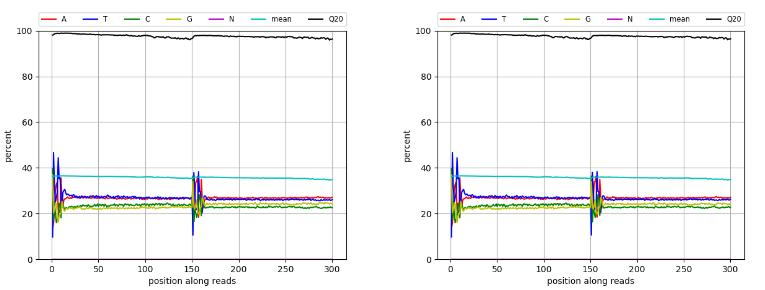


C

pre-filter after-filter

**Supplementary Figure 1.** Comparison of bases content along reads(pre-filter and after-filter) during varieties of myogenic differentiation stages. (**A**): The Q20 % and the GC concent (%) of early stage of differentiation (Day-0); (**B**): The Q20 % and the GC concent (%) of middle stage of differentiation (Day-3); (**C**): The Q20 % and the GC concent (%) of mature stage of differentiation (Day-7)
